# X-ray Imaging Analysis of Silo Flow Parameters Based on Trace Particles Using Targeted Crowdsourcing †

**DOI:** 10.3390/s19153317

**Published:** 2019-07-28

**Authors:** Andrzej Romanowski, Piotr Łuczak, Krzysztof Grudzień

**Affiliations:** Institute of Applied Computer Science, Lodz University of Technology, 90924 Lodz, Stefanowskiego 18/22 str., Poland

**Keywords:** measurement data analysis, targeted crowdsourcing, flow investigation tool, X-ray process tomography, radiography imaging

## Abstract

This paper presents a novel method for tomographic measurement and data analysis based on crowdsourcing. X-ray radiography imaging was initially applied to determine silo flow parameters. We used traced particles immersed in the bulk to investigate gravitational silo flow. The reconstructed images were not perfect, due to inhomogeneous silo filling and nonlinear attenuation of the X-rays on the way to the detector. Automatic processing of such data is not feasible. Therefore, we used crowdsourcing for human-driven annotation of the trace particles. As we aimed to extract meaningful flow parameters, we developed a modified crowdsourcing annotation method, focusing on selected important areas of the silo pictures only. We call this method “targeted crowdsourcing”, and it enables more efficient crowd work, as it is focused on the most important areas of the image that allow determination of the flow parameters. The results show that it is possible to analyze volumetric material structure movement based on 2D radiography data showing the location and movement of tiny metal trace particles. A quantitative description of the flow obtained from the horizontal and vertical velocity components was derived for different parts of the model silo volume. Targeting the attention of crowd workers towards either a specific zone or a particular particle speeds up the pre-processing stage while preserving the same quality of the output, quantified by important flow parameters.

## 1. Introduction

Crowdsourcing is an emerging method for processing large amounts of data using geographically-distributed heterogeneous workers. This method has proven suitable for resolving computational problems that are difficult to solve automatically, as it is capable of coupling the data processing capabilities of automated systems with human intelligence. Another advantage of crowdsourcing is the reduced cost (in terms of money, resources, or time). Most of the tasks submitted to widely-available crowdsourcing servers (www.mturk.com, www.crowdflower.com, www.crowdmed.com) could conceivably be processed by computer systems. However, due to their complexity or uniqueness, the difficulty of achieving sufficiently high accuracy, or simply economic factors, different methods of processing such datasets are preferred, based on human orientation. Examples of such tasks, including projects pertaining to image processing, have been reported in the literature [1,2,3]. One interesting example is the distributed diagnosis carried out by the medical community around the world, based on medical records submitted by patients who wish to obtain a second opinion. Another is the simultaneous translation or group editing of texts on-the-go, in which human resources can compete with automatic systems [4].

Crowdsourcing facilitates the delegation of work, which usually requires the use of Internet-connected computers [5]. An open call is made for people to contribute to online tasks that do not require any special knowledge, competences, or specific training in the field. Here, the crowd refers to an undefined, but large group of participants. It turns out that most mundane tasks involved in analyzing large datasets, especially imaging data, can be broken down into chunks that are able to be processed or annotated by an average person, without expertise in a particular domain or knowledge of the experiment.

Generally, there are dedicated platforms for managing the work distributed among individual workers. Entire jobs are divided into either smaller or sub-tasks in order to facilitate the process and achieve greater efficiency. Classical categorization into macro- and micro- (or sub-) tasks is a common part of crowdsourcing methods and is possible using most popular platforms [6,7]. However, most studies focus on dividing a sequence of consecutive data elements (usually image frames) into a smaller series of adjacent frames [8]. Each entire frame is then processed by an individual worker [9]. To the best knowledge of the authors, there have been no papers describing methods that target either only selected, limited areas of individual frames or single elements within these frames (or single elements visible on particular images); although, this is technically possible with the aid of existing platforms [7].

Crowdsourcing systems coordinate the efforts of distributed workers and synthesize the results to solve problems that a single individual could not achieve on the same scale, within the same budget or time-frame. This approach makes it possible to complete problems at a level of complexity beyond the capabilities of the research personnel available. Usually, a few minutes of online training in the form of a video or illustrated manual is sufficient for a new worker to begin the task. This is important from the point of view of this research, since neither machine learning algorithms nor standard image processing methods can currently perform efficiently in the application presented here, as reported in [10,11]. On the other hand, there is a number of limitations to the crowdsourcing method, as reported especially in business contexts, but affecting research projects as well [12]. One of the most important is the so-called sloppiness of the output [13]. This may result from a range of causes, one of which is the mundane nature of the work [14].

Crowdsourcing tasks can be efficiently tailored to a range of problems relating to process tomography sensing, especially for reconstructed images. There have been previous reports of the successful application of the general method to annotate X-ray radiography data using non-expert workers [11]. This paper takes a further step, using the output results for further analysis and interpretation, in this case to obtain the vertical and horizontal components of flow velocity in silos.

The main contribution of this paper is to evaluate the approach on the basis of industrial tomographic measurement data, which was impossible to process automatically using currently-available approaches, in order to provide a meaningful quantitative description of the flow in terms of parameters. An additional contribution of this paper is the proposed method of a targeted crowdsourcing, in which each contributor is given a narrowed down task in comparison to the usual full-frame work assignment. This involved identifying the position of a single marker or a set of markers in a small, selected area of the images in a sequence of assigned frames. Such targeting of the workers’ attention aims to reduce the effort required from any single participant, whilst providing data of comparable or superior accuracy to datasets processed using classical crowdsourcing systems.

The rest of the paper is organized as follows. Section 2, Section 3 and Section 4 cover the background and details of the interdisciplinary research. Section 2 discusses important related work in the field of gravitational flow of bulk solids’ process measurements. Section 3 gives details of the experimental procedure for X-ray measurements and data-processing issues. Section 4 presents the basic crowdsourcing method and data processing workflow employed in this work. The results and directions for future research are discussed in Section 5 and Section 6. A summary and conclusions are provided in Section 7.

## 2. Related Work

This section introduces the two key elements of the research background, namely industrial tomography for process monitoring and the basics of the gravitational flow of bulk solids.

### 2.1. Industrial and Process Tomography for Understanding Process Behavior

In the context of industrial processes, tomography is closely associated with imaging, since images provide rich information concerning the monitored process [15,16,17]. Optical systems are the most common for the visualization of liquid flows. They provide an image of the process using CCD/CMOS cameras. Electrical process tomography [18,19,20,21,22], gamma tomography [23,24], magnetic resonance tomography [25], X-ray tomography [26], or ultrasonography [27] can also be applied for this purpose. Since different types of imaging system are characterized by different modality properties (depending on the kind of process to be monitored, the type of installation, and the laboratory or industrial environment), different measurement setups will be applied. Electrical Capacitance Tomography (ECT) is best suited for the visualization of highly-dynamic processes, in which direct contact with the flowing medium is not possible, where the installation is opaque, or when the cross-section of the installation is substantial in size. However, although sufficient for control purposes, ECT imaging gives low spatial resolution. To investigate processes in the laboratory environment, using small-scale installations, other tomographic systems may provide more valuable information. For instance, X-ray tomography is a standard choice in the field of material science for investigating the structure of materials. This kind of tomography enables the analysis of phenomena that occur at the nano- or micro-scales. There are also reports in the literature on the development of X-ray systems for the visualization of processes at much larger scales, based on the low absorption of X-ray photons by a medium [28].

X-ray tomography provides much higher spatial resolution than electrical process tomography, making it more convenient and reliable as a tool for investigating processes. However, detailed imaging in 3D requires a substantial number of projections around the scanned body. Guillard et al. [29] presents a different type of X-ray imaging, namely the use of stereo-radiography for velocity and fabric fields’ determination for vertically plane-symmetric flow of granular media. In contrast, the study presented in this paper shows a single X-ray radiography system in application to granular media. We demonstrate that based on 2D images (radiographs), the behavior of the material during flow can be followed by tracing the positions of the tracking particles in symmetric bulk flow.

### 2.2. Gravitational Flow of Solids

The gravitational flow of solid particles is relevant to a number of industrial processes, in which bulk solids are stored in silos. Such granular material may be of natural provenance (e.g., sands and gravels) or be generated in the process of extraction (e.g., stone breakage), as well as being deliberately processed (e.g., plastic granules). The storage of bulk material, whilst seemingly very simple, is actually a complex process requiring sophisticated analysis. The quality of material storage, as well as the ease, efficiency, and safety of unloading strongly depend on the method by which the silo is filled [30]. The initial packing density places pressure on the walls, while the grain size and diameter, as well as the direction of deformation in the granulate particle systems affect changes in the concentration of bulk material in various areas of the tank during unloading. An additional difficulty with analyzing flow is the impact of many changeable external factors, including humidity and temperature. These factors can affect the behavior of the material during the loading and unloading of silos.

Systems for measuring the process of handling granular materials and measuring the levels of silos are well established. Since the early 1960s, studies focused mainly on predicting the type of flow (i.e., mainly funnel or mass flow) and its changes [31], analyzing internal shear zones [32,33], determining fields and velocity of flow, as well as predicting the intensity of emptying silos [34]. Variable parameters include the container geometry (shape, structure), the properties of the material (particle size and shape, packing density, coefficient of friction), and external conditions (temperature, humidity). Combinations of these parameters result in different flow behaviors, especially causing mass flow (i.e., the homogeneous downward flow of bulk with relatively similar velocity) or funnel flow (i.e., forming a core funnel, with bulk flowing at the center and the rest of the bulk forming a stagnant zone) [30,35]. Measuring changes in the concentrations and levels of materials in silos, which affect both the accuracy with which the velocity and mass flow can be determined and hence the safety of silo operation, is still a matter for continuing research.

### 2.3. Trace Particle Tracking Method for Flow Investigation

In recent years, Particle Tracking Velocimetry (PTV) and Particle Image Velocimetry (PIV) have been used extensively to measure the translational velocity field of granular assemblies [36]. Various reports on applications such as in vibrated beds [37], rotating drums [38], avalanche or debris flows [39], or hoppers/silos [40] are available. There are recent interesting reports on using X-ray PTV methods using stereography for fluidized beds with low-absorption medium as well [41]. In general, PTV and PIV enable volumetric measurements within a fluid flow or visible surface planar measurements for particle assemblies. For fluid flows, it is possible to capture the movement of tiny (micro-)suspended particles. The actual calculation is conducted with the aid of image post-processing techniques and algorithms responsible for image segmentation, particle identification, spatial mapping, and temporal tracking throughout the image sequences. While PIV gives the movement of particle assemblies, PTV traces individual particles, but gives better spatial resolution with the same or lower computing effort. All of the above-mentioned studies concerned tracking spherical particles, except for one, which applied non-spherical tracer particles [42].

## 3. Sensing Equipment and X-ray Imaging

**Figure 1:**
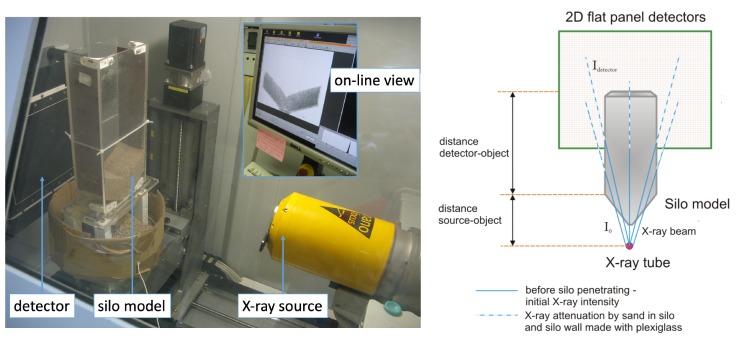
X-ray tomography imaging system: (left) photo of the silo model taken during X-ray scanning; panel detector on the far left, silo model on the left, X-ray tube on the lower right, and the corresponding online visualization included as an inset on the upper right; (right) schematic overview of the X-ray absorption model.

**Figure 2:**
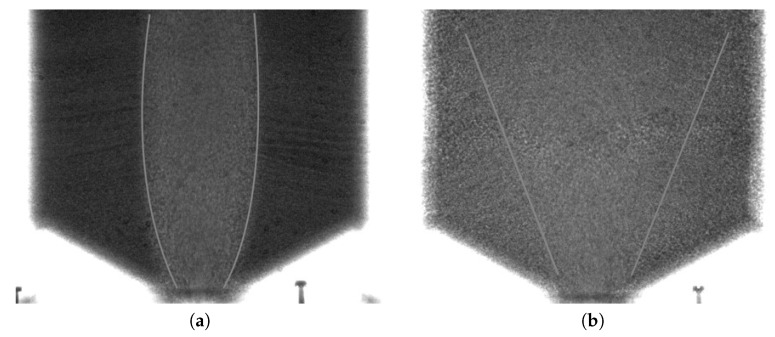
Radiography images of funnel flow in the silo: (**a**) initially dense packing; (**b**) initially loose packing. Contour lines indicate approximate flow funnel shape.

In order to investigate the two types of silo flow using an industrial X-ray tomography setup, a dedicated model of a silo was constructed. The rectangular silo model had 5 mm-thick walls made of perspex sheets, 340 mm in height, with an inner size of 150×70 mm (width × depth, respectively). The entire model consisted of an upper bin section and a lower hopper outlet section (photo on the left in Figure 1). The angle of the hopper from vertical was 60deg. The width of the rectangular outlet along the silo depth was 5 mm, and the length was 70 mm (as it was fixed along the entire depth of the model). Silo flow measurements were conducted at INSA-Lyon, MATEISCNRSlaboratory. We used a flat silicon panel detector tomograph produced by the Phoenix X-ray company. It was equipped with a Varian 2520V (PaxScanTM) detector made of a flat silicon panel, initially developed for medical applications. It was composed of 1920 rows and 1500 lines of sensitive pixels, each of which was 127×127μm${}^{2}$. The detector can be used in a 1×1 or in a 2×2 binning mode. During the measurements, the detector worked in the 2×2 binning mode (the pixel size was 254×254μm${}^{2}$). The X-ray source (a cone beam) was an open transmission nanofocus X-ray tube with radiation emission parameters of 145 kV (voltage) and 180 μA (current intensity). The source-detector distance was 0.577 m; the source-object distance and object-detector distance were 0.384 m and 0.193 m, respectively; and the magnification was 1.50. The geometric blurring magnitude was 2.01 μm. The exposure time was 100 ms, and the frame rate was equal to 10 fps. The beam hardening effect was counteracted using a thin copper plate (0.3 mm in thickness) mounted on an X-ray tube to filter the low energy X-rays. The resulting radiography size was 960×768 pixels. The captured sets of X-radiographs (for each experiment) were pre-processed, so as to extract and present information on changes in the X-ray radiation absorption coefficient. The X-radiographic image provided information on the X-ray attenuation within the object. Transmission radiography with a 2D flat panel detector was then used to generate a 2D map of μ, the linear attenuation coefficient. Figure 2 shows the two types of radiography images obtained. As we investigated two types of silo flow, the silo was filled on two separate occasions, which resulted in different measurement records and therefore significantly different reconstructed pictures of the flat panel detector output.

Trace particles (spherical metallic particles 2 mm in diameter) with relatively neutral buoyancy were mixed with a granular material (sand) before the experiments [43]. Initially, the particles were distributed randomly throughout the volume of the silo model. The intention was to observe their changing positions during flow, in order to derive information on the overall flow conditions in the container. By analyzing 2D radiographic images, it was possible to estimate the paths of particles moving in 2D space. To investigate the 3D nature of the flow, it would have been necessary to analyze a 3D tomography image. This paper focuses on 2D analysis only. The main reason for choosing 2D radiography is its much higher temporal resolution, which enables the measurement of dynamic processes, such as gravitational bulk flow. X-ray imaging in 3D would require many more measurements per time unit, and therefore is not suitable in this case.

One notable drawback of the applied imaging technique, however, lies in the flatness of the generated image. Some features of the observed process may have been concealed by objects closer to the emitter. Due to this “shadowing” effect, some of the objects may be partially visible (i.e., their shape is distorted and the area of the object is smaller than in reality) or totally invisible in certain random individual or consecutive frames. Such behavior must be taken into consideration in the subsequent analysis, as it causes issues for automatic processing of trace particle images using both classical image processing algorithms and modern approaches such as neural network-based methods [10].

### 3.1. X-ray Measurement Data Processing

Radiography images were taken of funnel flow in the cases of dense and loose initial packing densities. The flow area in each case was different, since for initially dense packing, the funnel should be narrower than in the case of loose packing. In addition, the contrast between the funnel zone and the remainder of the material in the radiography images was much higher in the case of dense flow. Figure 2 shows two radiography images for initially dense and loose packing densities. As may be imagined, analyzing a series of such images could result in different rates of eyestrain and hence different anticipated demands and cognitive workload. More details on the visualization of funnel flow can be found in [44].

**Figure 3:**
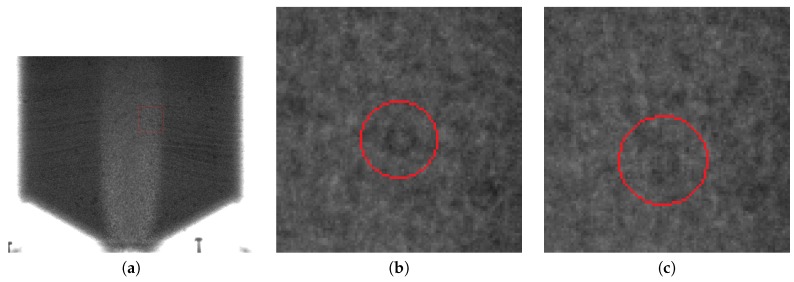
Difficulty of distinguishing markers in radiography images: (**a**) frame with the investigated area marked, (**b**) zoomed-in marker, and (**c**) the same marker on the next frame.

Figure 3 illustrates the difficulty of extracting the trace particles from the background noise. Due to limitations in both the spatial and temporal resolutions of the collected flow data, the markers were often distorted, smudged, or blurred to the point where they became undetectable by any morphological or shape-metric approach. Therefore, detecting the particles by automatic means is very problematic, and even for humans is extremely difficult.

The combination of inherent graininess at such scales and the grainy nature of the observed material itself probably makes extraction of the relevant features in the image an insurmountable task for classical image processing methods. However, as previously shown [11] and observed again in the course of this research, human operators can be taught to distinguish such markers in a relatively short time. Furthermore, they can attempt to estimate the approximate position of the marker given the previous or next frame. A human operator can also anticipate the existence of an object based on a wider temporal sequence and locate it even if it is significantly occluded by the surrounding material in one or more of the recorded frames. In this work, we investigated different approaches for using crowdsourcing to annotate images. We developed a simple application with a front-end graphical interface implemented in Python and Pyside2. With the aid of this tool, it was possible to rapidly distribute the raw datasets and assign tasks to separate workers.

### 3.2. Experimental Methodology

**Figure 4:**
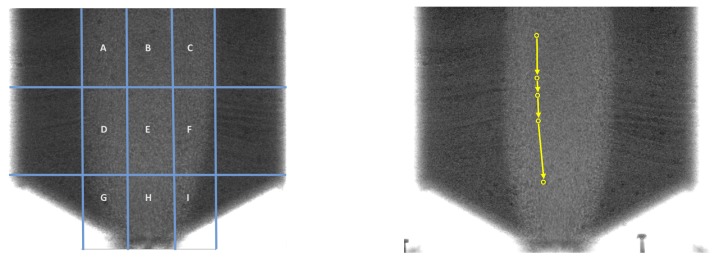
Comparison of the zone-targeted mode and single-particle-targeted method of crowdsourcing: (left) grid showing velocity calculation zones used as target zones in the zone-targeted tracking method; (right) illustration of single-particle-targeted method for tracking an arbitrary trace particle.

Downward velocity was calculated based on the positions of particles provided by the crowdsourcing system (details on the crowd workflow are given later in this paper). In order to compare the velocity for different heights above the silo outlet, the area of the silo was divided into nine sections. This division was established based on expert knowledge of the gravitational flow process, with each zone containing part of the funnel flow. Figure 4 shows an example zone grid, predefined to determine the velocity components. The funnel flow area contained three central zones (B, E, H) and six side segments (A, C, D, F, G, I). Analysis of the speed of the flow of loose material was carried out based on the established trajectories of trace particles found in the zones. The analysis was completed for both loose and dense initial packing densities.

Three crowdsourcing data processing methods were applied. The first method was an established classical procedure (the “classical” method), according to which each worker processed all the particles visible in the picture on each image in the assigned sequence. The second was a zone-particle tracking method, whereby each worker focused on and processed a limited number of trace particles located only within a single zone of each image in the assigned sequence. For this method (the “zone-targeted” method), we used the same zone grid as for the comparison of flow velocity, as shown on the left in Figure 4. Finally, a single-particle method (the “single-particle-targeted” method) was applied, in which each worker processed only one, selected trace particle, tracking it through consecutive images of the assigned sequence, ignoring all other particles. The picture on the right-hand side in Figure 4 illustrates the process of tracing a single particle in the single-particle targeted method.

The first, classical method was an implementation of the well-known crowdsourcing paradigm, while the other two (i.e., targeted zone and single-particle methods) were novel and are evaluated within this study. Both require an additional initialization step, which involved preparing (marking) the first frame of the assigned image sequence in order to indicate the zone of interest (for the targeted zone method) or either selecting the starting particle or giving instructions on how to select the particle of interest (for the single-particle-targeted method).

## 4. Crowdsourcing as an Effective Image Processing Engine

### 4.1. Crowdsourcing System Workflow

**Figure 5:**
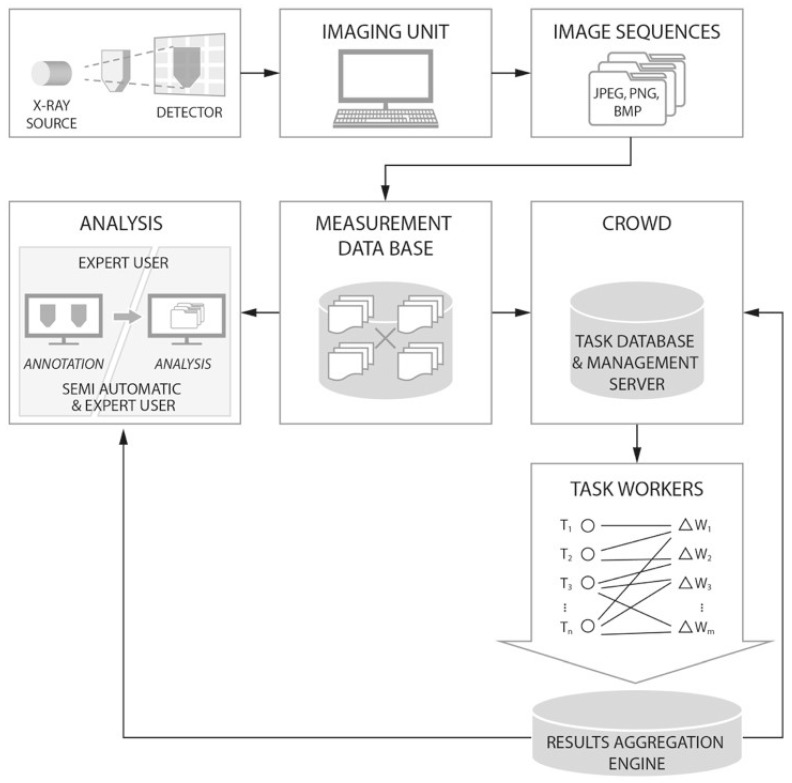
Complete workflow diagram of the data processing and analysis system for tracking particles during bulk solid flow monitored by X-ray imaging.

The general workflow for processing the X-ray trace particle images is presented in Figure 5. The solution employed neither a dedicated system, nor a high capacity big data framework, yet the modular design supported easy extension [45,46]. First, radiography images captured by the X-ray imaging device were transferred to a common database, as shown on the top row of the figure. A measurement database (as represented at the center of the middle row, Figure 5) provided the source of the data that could be used by the operator (either a human operator or automatic system) to make decisions regarding further processing. The next possible steps were (#1) to employ an expert to analyze the data visually (analysis module on the left-hand side of the middle row, Figure 5) or (#2) to send them to the crowdsourcing system (right-hand side in the middle row, Figure 5) for distribution and processing (bottom row, Figure 5).

Option (#1) was a typically mundane task. The volumetric parameters of the flow were the required output. Suppose there were 1000+ images in sequence, showing up with 50 tracking particles. At least those in the center (usually 20+ particles) needed to be analyzed in each picture. The analysis consisted of tracing the movement of the particles in consecutive frames. Heterogeneous damping of radiation resulted in non-uniform representation of the tracing particles on adjacent images, which created difficulties for automatic image processing algorithms to detect them efficiently, as described previously in Section 3.1. Due to the fact that this was a highly repetitive procedure, a gradual drop in the quality of the obtained parameters was to be expected [47].

**Figure 6:**
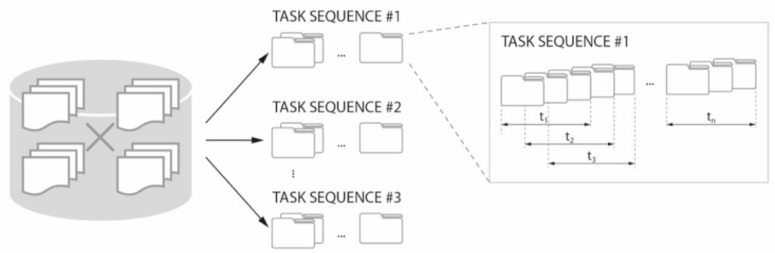
Schematic algorithm for task replication and dispatching in the crowdsourcing system.

Option (#2) involved uploading selected datasets to a crowdsourcing system for distribution among workers. Different strategies for dividing, managing, and verifying the results are used in commercial crowdsourcing systems. However, a simple approach was used in this work [12]. The task of each worker was to process a sequence of frames (images), always showing a fixed, predefined fragment of the full experimental sequence (for example, workers may process sequences of several tens of frames, up to 100 frames). Since each frame needed to be annotated in such a way as to mark all the trace particles that can be distinguished by a human on a single image, each complete annotation may consist of multiple sub-annotations, carried out by different volunteers on partially- or fully-overlapping areas of the frame. The positions of the markings (the central coordinates of the sphere) were recorded for each particle and each frame. Usually, many workers process the same frame frame sequence fragments. Hence, these fragments may overlap. However, these overlapping fragments did not have to be the same frame fragments, nor did the frame sequence lengths processed by different workers have to be equal, as the start and end points of the sequence fragments may vary (as illustrated in Figure 6). The proposed targeted crowd sourcing approach introduced the additional condition of processing only a confined, limited part of each frame. Yet, this did not interfere with the described procedure, and different areas of focus within the frames may be assigned to different workers. We applied both methods (full-frame and partial processing) in this work, and a comparison of the results is presented later in the text.

When the worker finished with a single frame, the marked particles were saved and the markings transferred to the next picture in the sequence. In the next picture, the worker could adjust the positions of the markings indicating the particles, since generally there were only slight shifts between the particles on consecutive images as the bulk flow proceeded. The markings from the previous frames were reported to be helpful, as the trace particles may not be equally exposed on consecutive images [48]. Workers could remove the markings that no longer belong to any trace particle on the current picture in the sequence. Once the fragments were processed, the results (the coordinates of the annotated particles) were aggregated, as illustrated in the bottom row of Figure 5. The average coordinates were calculated, based on superposition of the trace particles marked by different workers. Some of these results (especially for the first frames) were transferred back to the management database until all the scheduled tasks had been completed. In the final step, as illustrated in Figure 5, the output of the crowdsourcing was fed into the interpretation stage. This was where it was analyzed by the domain expert or, ideally, treated as an input for an automatic support system used by the expert. In the latter case, it could be further processed based on the numerical data received from the crowdsourcing system. Otherwise, it was possible for the expert to analyze the images previously annotated by the crowd workers directly.

We used three different approaches (full-frame, zone targeted, and single-particle targeted), as described in Section 3.2. However, none of those different methods affected the overall crowdsourcing data workflow. Only minor changes to the initial training given to the workers before starting the task were necessary in order to instruct them on how to proceed in each particular mode.

### 4.2. Crowdsourcing System Output

**Figure 7:**
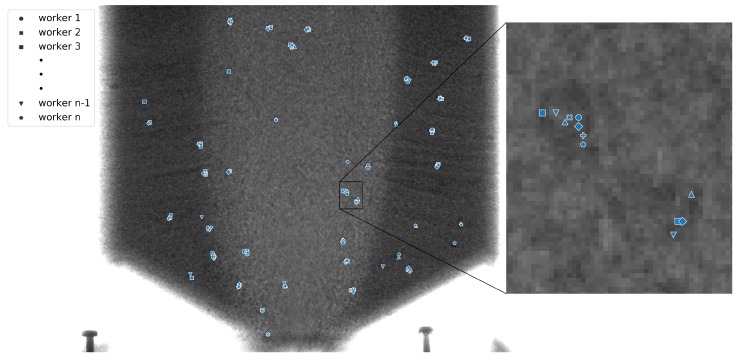
Example of the annotated X-ray image with noticeable discrepancies between the labels positioned by different workers. (Left) Full image view. (Right) Zoom-in showing slight shifts between the centers of markers set by different workers.

The output of the crowdsourcing system was two-fold. Firstly, it exported images with the tracked particles marked (annotated) on each frame. The aggregated trajectories of the particles may also be marked. Secondly, numerical data conveying the exact positions of each particle in each frame were exported from the system. Figure 7 presents the results of the crowdsourced annotation of a sample frame. It is worth noting that whilst each user was generally capable of placing the label on the trace particle, there was some discrepancy between the exact locations of the markers. Hence, these labels needed to be aggregated in order to obtain their final positions. This aggregation can be carried out either using statistical methods or, should the required end result be only visual, through simple morphological operations.

There are different approaches to the process of annotating the images. The usual way is to mark the region of interest with a circle of a specific, highly-contrasting color, or to place a marker at the approximate center of the region [11]. Some other designs of the user interface utilize a range of colors that convey additional information, such as the marking already processed in the current or previous frames, shifts from previous positions, etc. [48].

## 5. Results and Discussion

### 5.1. Flow Velocity Determination

The first goal of this work was to test crowdsourcing as a valid method for analyzing experimental radiography data, supporting the determination of meaningful process parameters. Therefore, a quantitative scenario for calculating the velocity of each marked particle based on the position extracted from the system output was designed. A pilot study was conducted with two domain experts and up to n=22 distributed participants processing the assigned fragments as crowd workers. The workers were presented with a series of chunks taken from experimental datasets showing silo flows. The lengths of the chunks ranged from 10–140 frames, but typically contained at least 40 frames. For calculations, we used the superimposed positions of between seven and 19 individual trace particles, overlapping on separate frames. In order to compare flow for different conditions (dense, loose) and for different heights, particles located at the center of the silo were chosen. This approach reduced the displacement of particles from the main path of the flowing particle. The following four tables present calculated velocity results for each silo zone for initially dense (Table 1 and Table 2), as well as loose (Table 3 and Table 4) packing density conditions. Table 1 and Table 3 give an overview of the obtained numbers in pixels per frame, while Table 2 and Table 4 give the actual calculated velocity in mm per s. Intermediate state results are shown in pixels in order to give the reader a deeper understanding of the consecutive steps in the algorithmic procedure of using crowdsourcing for velocity determination and to demonstrate its consistency.

Table 1, which presents initially dense packing conditions, shows the four components of velocity: Vx: horizontal Velocity (upper left corner), Vy: vertical Velocity (upper right corner), Vt: total Velocity (bottom left corner), Vp: pointer index Velocity (bottom right corner); while Table 2 gives a simplified view of Vx and Vy only, but given in mm/s. Table 3 and Table 4 give similar results for initially loose packing conditions, accordingly.

**Figure 8:**
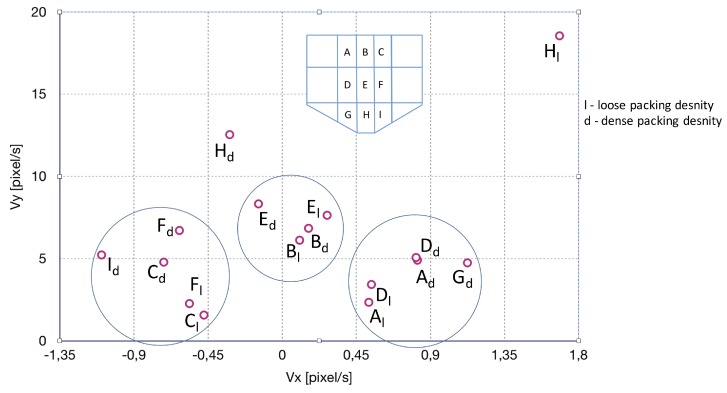
Trace particle-based silo flow velocity distribution map. Small circles show velocity values for distinct silo zones (from A–H), as indicated on a schema drawn in the upper part of the picture. Large circles show groups of similar results. The lower index d indicates the initially dense packing condition, while index l indicates the initially loose packing condition.

As anticipated, the higher overall velocity was observed in the center zones of each row (B, E, H) than in the side zones (A, D, G, C, F, I). The highest velocity was observed at the bottom of the hopper component of the silo: Zone H [34,49]. The comparison of dense and initially loose packing density flow, based on velocity component analysis (Table 2 and Table 4), produced a significant difference in Zone H, up to 50% in the overall velocity values. While the central zones for both conditions were similar (Zones E, B), the side zones (Zones D, F, A, C) differed (by more than 65% for Zones C and F), since the character and shape of the funnel varied. The increasing differences between the initially dense and initially loose packing conditions may be explained primarily by the varying size of the flow area (funnel area; see Figure 2). The wider the funnel, especially in the upper part of the silo (Zones A, B, C), the greater the difference (assuming the silo outlet is the same size). These differences were also visible in the values of horizontal component velocity, where in the case of initially loose packing density, the absolute values inside of side zones were generally higher than for initially dense packing density (Zones A, C, D, F). Figure 8 shows a velocity distribution map derived on the basis of the results obtained. Groups of similar velocity vectors were arranged in circles. It can be noted that similar results were obtained for the three main vertical Zones, A, D, and G (left of the funnel), C, F, and I (right of the funnel), and finally, B and E (upper center of the funnel). These results were consistent regardless of the condition, i.e., they were similarly situated on the velocity map for both loose and dense initial packing densities. The only exception was the velocity for H, which was the lowest funnel zone, just above the outlet where the particles gained the highest velocity. Therefore, given that the results for $H_{d}$ (dense) and $H_{l}$ (loose) were still within a moderate range of values conforming to theoretical expectations, the efficacy of the method was proven.

The results presented here were for the single-particle method, but the accuracy and precision calculated for all three methods were satisfactory and did not differ significantly. First of all, we considered accuracy compared to the ground truth baseline prepared by two experts. All the methods achieved comparable results, with variations of no more than 2%. Precision taken as the repeatability of results also reached 98%. Next, we examined the duration, i.e., the length of time required to complete the task by workers using different methods.

Table 5 shows aggregated times taken by workers to complete the tasks. The columns show the results for classical, zone, and single-particle methods, respectively. The rows show average results for processing a single frame, 10 consecutive frames, or 100 frames (from top to bottom, respectively). SD indicates the Standard Deviation. The single-particle targeted method was the fastest, as was to some extent expected. However, it should be noted that it consumed approximately 10-times less time than the classical method and almost four-times less time than the zone particle method. These factors were greater than anticipated, since there were no cases in which there were 10 trace particles; the average maximum number oscillated around four or five for most of the populated zones. The zone particle method was more than twice as fast as the classical method in all cases. Given the ability of crowdsourcing to parallelize jobs, it may be possible to speed up the entire process significantly. It is also worth noting that the SD was significant, since some frames or frame sequences were much more difficult to process (or simply required more time to process).

Table 6 shows average processing times for different zones. The most important zones were the central funnel flow zones, i.e., B, E, and H. However, no significant differences were visible, since both the processing time and SD remained close to the average values.

By quantitative analysis of radiographic images with the aid of the crowdsourcing system, it is possible to obtain a profile of the granular material velocity during the silo discharging process. The results provided additional knowledge about granular flows, making detailed comparative analysis of flow dynamics possible. Such analysis can be conducted on the basis of the calculated velocity profile derived from X-ray imaging data. The results obtained in our study are in agreement with previously-reported data [34,49].

The proposed crowdsourcing system enabled the distribution of the imaging data (image sequences) for different flow fragments (see Figure 6 for the task allocation algorithm). The results showed better quality particle detection for frames pre-marked based on previous images in the sequence. More details on the development of the crowdsourcing method and system were given in [11,48]. In contrast, workers reported decreasing efficiency due to rising fatigue related to physical and cognitive workload demands over time when they worked with longer fragments of image sequences. Therefore, in the future, it would be interesting to investigate whether it would be beneficial to work with the system at random times chosen by the workers, of limited durations, possibly adjusted to the specific needs of the workers. Further development of the system itself, as well as of the crowdsourcing methodology for tomographic imaging analysis will be continued in the next stages of this research.

### 5.2. Qualitative Assessment: NASA TLX

In order to assess the workload of the participants, we performed NASA Task Load Index (TLX) tests. The participants completed a self-assessment rubric, in which they evaluated six main factors related to the given tasks, namely mental, physical, and temporal demand, how they perceived their performance in terms of quality and effort, and finally, the level of frustration induced by the task. These factors approximated to some extent the measurement of task complexity, the user experience, and the usability of the proposed approach, all in relation to the background of individual workers.

**Figure 9:**
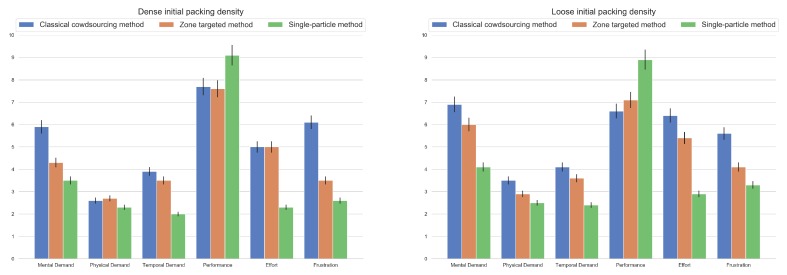
NASA Task Load Index (TLX) plot for the classical method (blue bar, left-hand side in each triple post), zone-targeted crowdsourcing method (orange bar, middle position in each triple post) and the single-particle focused crowdsourcing method (green bar, right-hand side in each triple post) for two initial silo filling conditions, i.e., dense (left) and loose (right) initial packing density.

Diagrams of the TLX results are presented in Figure 9, separately for the two conditions for the processed X-ray measurement results, i.e., dense silo filling (on the left-hand side) and loose silo filling (on the right-hand side). The colored bars for each TLX category represent results obtained using the three methods. The blue bar (always on the left in each group) shows the performance of the baseline crowdsourcing method, i.e., results achieved by crowd workers annotating all the trace particles in each frame. The orange bar (always in the middle) illustrates the performance of the zone tracking method, i.e., results achieved by crowd workers annotating trace particles bounded by a single area, as depicted in Figure 4. The green bar (always on the right) illustrates the performance of the single-particle tracking method, i.e., results achieved by crowd workers annotating only a single particle of their choice, taken from a particular, indicated area.

The NASA TLX index tests showed a significant decrease in mental and temporal demand, as well as a drop in job frustration for both conditions (dense and loose silo filling), for both proposed methods compared to the classical crowdsourcing method. The results were better (a larger drop) for the single-particle tracking method than for the zone-tracking targeted method. However, the decrease in physical demand was slightly more prominent for the single-particle method and was significant for both methods only in the case of the loose filling condition. A different effect can be observed in the category of effort. Effort was reported to decrease significantly, mainly for the single-particle method (a drop of more than 50%) compared to the classical baseline method. Performance went up significantly, by more than 10%, but only for the single-particle method. Interestingly, performance increased by more than 10% only for the single-particle method. Performance was not perceived to be significantly different for the zone-tracking method, no matter the condition, yet it was perceived to be worse than the classical method in the case of dense filling.

### 5.3. Discussion Summary

The methodology presented here provides a practical way to analyze reconstructed images, when automatic methods for feature extraction using classical computer vision algorithms are not efficient. The proposed method based on crowdsourcing was verified using real measurement data, and the results were in agreement with those obtained by other research methods. Specifically, the velocity vectors calculated using the crowdsource-annotated data were verified. The positions of trace particles were annotated in sequences of radiography data over time and were used to determine velocity vectors in three different parts of a silo container during bulk solid flow. In this paper, we showed a method for how to determine the flow velocity based on horizontal and vertical velocity components of trace particles.

In our comparison of three approaches to crowdsourcing the processing of image sequences, the proposed zone-targeted and single-particle targeted methods performed well, giving task completion time benefits. Further investigation is needed to prove the usefulness of the methodology for other applications.

## 6. Future Work

Our results showed definite potential for further applications of the targeted crowdsourcing methods. Methods that decrease overall workload are needed to reduce common limitations of crowdsourcing, such as sloppiness and the low percentage of quality results [13,14]. More research is required on how to apply these targeted methods to different tomography sensing problems, which are difficult to process and interpret automatically. Future work could also attempt to couple these methods with novel Virtual Reality (VR), Augmented Reality (AR), and Mixed Reality (MR) visualization technologies [50], in order to design novel interfaces for future-of-work and Industry 4.0 mash-ups of human operators working in AI-driven automated industrial process installations [51,52]. It is worth emphasizing that the output of the crowdsourcing methods is a labeled dataset that could later be used as input data for the further training of machine learning algorithms. Given a large enough dataset, it is anticipated that the process could be automated, at least to some extent, for classes of similar images.

## 7. Conclusions

In this work, we presented a method for extracting process parameters using crowdsourcing. The proposed data processing workflow was applied to study gravitational silo flow, measured by X-ray radiography, and proved to be a reliable and useful way to process tomography data. Crowdsourcing proved to be efficient for pre-processing raw images captured by an industrial standard 2D X-ray radiography sensor. The aggregated average output from the crowdsourcing system can be taken as the input for further, automatic or semi-automatic calculations, as demonstrated here in the case of calculations of the axial velocity of trace particles moving along a silo during unloading. An additional benefit of the proposed targeted crowdsourcing method is that it minimizes the cognitive workload, enabling similar research tasks to be completed more efficiently.

## Figures and Tables

**Table 1:** Velocity components for dense initial packing density presented for nine zones, from A–I. Each quadrant presents Vx (V, velocity), Vy, Vt (t, total), or Vp (p, pointer), clockwise from the upper-left corner. Vx, Vy, and Vt are given in pxpf (pixels per frame).

0.8213	4.9124	0.1602	6.8481	−0.7188	4.7917
A		B		C	
4.9806	16.49%	6.8500	2.34%	4.8453	−14.83%
0.8131	5.0736	−0.1438	8.3339	−0.6240	6.7173
D		E		F	
5.1383	15.82%	8.3352	−1.73%	6.7462	−9.25%
1.1250	4.7500	−0.3187	12.5440	−1.09651	5.2293
G		H		I	
4.8814	23.05%	12.5480	−2.54%	5.3430	−20.52%

**Table 2:** Velocity components for dense initial packing density presented for nine zones, from A–I. Each quadrant presents horizontal (Vx on the left) and vertical (Vy on the right) components only. Vx and Vy values are given in mm/s.

1.3901	8.3142	0.2711	11.5905	−1.2165	8.1099
A		B		C	
1.3762	8.5871	−0.2434	14.1052	−1.0562	11.3690
D		E		F	
1.9041	8.0394	−0.5394	21.2306	−1.8558	8.8506
G		H		I	

**Table 3:** Velocity components for loose initial packing density presented for seven zones, from A-F and H (Zones G and I show no results for the loose condition since they were outside the funnel). Each quadrant presents Vx, Vy, Vt, or Vp, clockwise from the upper-left corner. Vx, Vy, and Vt are given in pxpf (pixels per frame).

0.5263	2.3509	0.1062	6.1258	−0.4750	1.5750
A		B		C	
2.4091	21.85%	6.1267	1.73%	1.6451	−28.87%
0.5415	3.4333	0.2735	7.6415	−0.5631	2.2733
D		E		F	
3.4758	15.58%	7.6464	3.58%	2.3420	−24.04%
		1.6844	18.5511		
G		H		I	
		18.6274	9.04%		

**Table 4:** Velocity components for loose initial packing density presented for nine zones from A–I (zeros for G and I). Each quadrant presents horizontal (Vx on the left) and vertical (Vy on the right) components only. Vx and Vy values are given in mm/s.

0.9808	3.9789	0.1797	10.3679	−0.8039	2.6657
A		B		C	
0.9165	5.8109	0.4629	12.9332	−0.9530	3.8475
D		E		F	
0.0000	0.0000	2.8509	31.3978	0.0000	0.0000
G		H		I	

**Table 5:** Comparison of annotation time for different crowd work strategies. Time values are averaged and rounded to two decimal places and given in s.

	Classical		Zone Targeted		Single-Particle Targeted	
	Time (avg)	SD	Time	SD	Time	SD
1 frame	58.47	36.26	18.57	15.13	5.94	4.20
10 frames	584.69	262.51	185.70	121.65	59.41	12.30
100 frames	5800.53	1952.39	1845.58	978.37	591.57	68.43

**Table 6:** Comparison of annotation timed for different zones. Average times rounded to two decimal places and given in s.

Zone	Time	SD
A	9.60	2.70
B	13.33	3.67
C	47.07	18.24
D	9.11	3.62
E	19.04	6.38
F	8.56	5.18
G	12.80	4.62
H	15.66	4.92
I	31.98	15.78
